# Overview of the National Institute on Aging

**DOI:** 10.1093/geroni/igaa057.3025

**Published:** 2020-12-16

**Authors:** Richard Hodes

**Affiliations:** National Institutes of Health, Bethesda, Maryland, United States

## Abstract

Dr. Hodes will provide an overview of National Institute on Aging research priorities and funding availability.

